# Case Report: Fatal case of dual infection *Metapneumovirus* complicated by *Streptococcus pyogenes*

**DOI:** 10.3389/fmed.2025.1576583

**Published:** 2025-04-22

**Authors:** Danil Krivonos, Alexander Pavlenko, Alexandra Lukina-Gronskaya, Elena Korneenko, Anna Speranskaya, Elena Ilina

**Affiliations:** ^1^Research Institute for Systems Biology and Medicine (RISBM), Moscow, Russia; ^2^Moscow Center for Advanced Studies, Moscow, Russia; ^3^Saint Petersburg Pasteur Institute, Federal Service on Consumer Rights Protection and Human Well-Being Surveillance, Saint Petersburg, Russia

**Keywords:** intoxication, bacterial infection, *Streptococcus pyogenes*, *Metapneumovirus*, sequencing

## Abstract

Human *Metapneumovirus* (hMPV) is a common cause of acute respiratory viral infection in humans, typically occurring in children and causing no serious complications. However, the severity of the disease can be exacerbated by certain bacterial pathogens that lead to severe illness and even death. This report details a fulminant case of dual infection with hMPV and group A *Streptococcus* (*Streptococcus pyogenes*) in a three-year-old child. The whole genome sequencing of isolated clinical *S. pyogenes* strains was conducted, followed by an analysis of the genomic characteristics of the pathogen. Also, potential viral and bacterial pathogens were identified by qPCR and 16S rRNA metagenomic sequencing in any autopsy materials obtained from the patient. Children who had contact with the patient and began to exhibit symptoms of a cold were also tested and confirmed to have uncomplicated hMPV infection. The *S. pyogenes* strain has been found to contain five genes for various streptococcal exotoxins (*speA, speB, speJ, speG* and *smeZ*). In addition, the *speA* gene is situated in close proximity to the prophage, which may suggest that it is encoded and transferred specifically by the bacteriophage. We hypothesize that it was the cumulative effects of different streptococcal exotoxins that led to the patient’s death.

## 1 Introduction

Human *Metapneumovirus* (hMPV) is a prevalent infection affecting both adults and children. Nevertheless, the majority of cases of hMPV occur in children under the age of 5 years (1). The incubation period of the virus is usually 3 to 5 days. Patients may present with a variety of symptoms such as fever, cough, hypoxia, upper respiratory tract infection, lower respiratory tract infection and wheezing (2). The majority of individuals infected with hMPV do not experience significant complications. Nevertheless, in certain instances, the illness can be quite severe and even fatal. In particular, the presence of an additional bacterial infection can result in fatal outcomes. There are numerous documented instances wherein a bacterial infection arises concurrently with a hMPV infection, resulting in a more severe clinical course. In addition, there are numerous instances of a combined infection involving a hMPV and a bacterial pathogen belonging to the genus *Streptococcus* (1,3,4). This can result in a general deterioration in the patient’s condition and intoxication.

Such conjugate infections are more dangerous for children, whose immune systems are still developing. This paper presents a case of dual infection with hMPV and group A *Streptococcus* (*Streptococcus pyogenes*), which resulted in death. In this paper we analyzed the whole genomes of isolated *S. pyogenes* strains and as well as microflora composition in contact persons.

A notable feature of this case is the absence of obvious signs of bacterial infection in the patient, which meant that there were no obvious indications for antibiotic therapy. At the same time, *S. pyogenes* caused severe intoxication in the patient, which ultimately led to death.

The incident in question occurred in November 2022 in the Leningrad Region of Russia. A three-year-old child, male, was admitted to the hospital with complaints of increased body temperature for 2 days, vomiting 3 times a day, liquid stools 3–5 times a day, and a barking cough. The patient became acutely ill 2 days before hospitalization. He had a runny nose, fever up to 38.5°C, and a barking cough. On the day of the disease the patient was examined by a pediatrician, inhalations with pulmicort, ingaverine, and irrigation of the pharynx were prescribed. Previously, the patient had no such complaints. The patient was admitted in serious condition with stage 3 respiratory failure, intoxication syndrome, and a temperature 35.9°C. Upon examination, hypertrophied and hyperemic tonsils were revealed, without plaque, the lymph nodes were not enlarged. Breathing through the nose is free with bloody discharge, shortness of breath is mixed, and the pulse is rhythmic at 168 per minute. When breathing, the right half of the chest lags behind, the respiratory rate is 52 per minute, breathing with the participation of auxiliary muscles. Saturation is 72% without additional oxygen and 82% with oxygen. A chest X-ray showed signs of right-sided total pneumonia. Due to increasing respiratory failure, the child was placed on artificial ventilation with inotropic support with norepinephrine. After 2 h, cardiac arrest is recorded. Cardiopulmonary resuscitation is performed according to the ACLS protocol. An hour later, the patient dies. The cause of death according to the results of the pathological examination was endotoxic shock R 57.8 ICD, consumption coagulopathy D 65.X ICD, pneumonia due to other specified infectious organisms J 16.8 ICD, and other specified immunodeficiencies D 84.8 ICD.

In the general blood test, leukopenia was observed (3.7 × 10^9^ cells/L, norm 5–12 × 10^9^ cells/L), thrombocytopenia (153 × 10^9^ cells/L, norm 210–490 × 10^9^ cells/L), a decrease was observed in the leukocyte count the proportion of segmented neutrophils (13%, normal 41–43%), monocytes (1%, normal 8–10%), an increase in the proportion of lymphocytes (79%, normal 40–45%). In a biochemical blood test, an increase in the level of ALT (99 U/L, norm 0–29 U/L), AST (132 U/L, norm 0–36 U/L), CRP (102.1 mg/L, norm 0 is observed. -5 mg/L), urea- (12.7 mmoL/L, normal 3.8–7.3 mmoL/L), creatinine (148 mmoL/L, normal 80–115 mmoL/L).

Children who were in contact with the patient (hereinafter referred to as contact persons) also had some cold symptoms, which are summarized in Table 1. The contact persons were children from 3 to 8 years of age from the common group with the patient, and 13/13 had positive PCR tests for hMPV. To gain a more detailed understanding of the similarities in the disease course, nasopharyngeal swabs were collected from contact persons (CP1, CP3, CP4, CP6, CP7_2, CP8, CP9, CP15, CP7, CP17, CP19, CP57, CP58) and delivered to the laboratory for analysis. The autopsy materials from the patient himself were delivered to the laboratory, including the right lung (K28313), the left lung (K28314), the trachea (K28315), and the spleen (K28316).

**Table 1:** Results of analysis of samples received from the patient and from contact persons.

Sample	Clinical picture	Source	Viral infections (RT-PCR analysis)	Bacterial infections (RT-PCR analysis)
K28313	Cough, diarrhea, normal body temperature, intoxication, pneumonia	RL	hMPV	*S. pyogenes*
K28314		LL	hMPV	*S. pyogenes*
K28315		Tr	hMPV	*S. pyogenes*
K28316		Sp	PCR failed	*S. pyogenes*
CP1	Right-sided segmental pneumonia, cough, normal body temperature	Ns	hMPV	Neg.
CP3	Laryngitis without stenosis, barking cough	Ns	hMPV	Neg.
CP4	Bronchitis, cough, normal body temperature	Ns	hMPV	*S. pneumoniae*
CP6	Wet cough, Normal body temperature	Ns	hMPV	*S. pneumoniae*
CP7_2	Cough, normal body temperature	Ns	hMPV	Neg.
CP8	Pharyngitis, rare cough, normal body temperature	Ns	hMPV*, Adenovirus,* Epstein–Barr virus (EBV)	Neg.
CP9	Acute bronchitis, cough, normal body temperature	Ns	hMPV	*S. pneumoniae*
CP15	Bronchitis, cough, normal body temperature	Ns	hMPV	Neg.
CP7	Rhinorrhea	Ns	hMPV	Neg.
CP17	Sore throat, nasal congestion	Ns	hMPV*, Adenovirus*	*S. pneumoniae*
CP19	Asymptomatic	Ns	hMPV*, Adenovirus, Rhinovirus*	Neg.
CP57	Acute bronchitis, cough, subfebrile temperature	Ns	hMPV	Neg.
CP58	Nasopharyngitis	Ns	hMPV	Neg.

Source for microbial analysis. RL, Right lung tissue; LL, Left lung tissue; Tr, Trachea tissue; Sp, Spleen tissue; Ns, Nasopharyngeal swab.

## 2 Materials and methods

### 2.1 Pure culture isolation

Isolation of *S. pyogenes* from autopsy specimens was performed using Columbia blood agar (Himedia Laboratories Pvt. Ltd., India) supplemented with 5% bovine blood. Identification of *S. pyogenes* was done by MALDI-ToF mass spectrometry using the BactoSCREEN analyzer (Lytech Ltd., Russia). Genomic DNA of *S. pyogenes* was isolated by bacterial DNA extraction kit (Biolabmix, Russia).

### 2.2 DNA manipulation

The isolation of total DNA/RNA from autopsy samples was done using the RIBO-prep Kit (AmpliSens, Russia) in accordance with manufacturer’s instruction.

The isolation of nucleic acids from nasopharyngeal samples was conducted using the MagMAX™ Microbiome Ultra Nucleic Acid Isolation Kit, with bead tubes and the KingFisher™ Purification System (Thermo Fisher Scientific, USA), in accordance with the instructions provided by the manufacturer.

The presence of genomic DNA or RNA of *Metapneumovirus*, *Rhinovirus*, *Adenovirus*, *Epstein–Barr Virus, Streptococcus pyogenes*, and *Streptococcus pneumoniae* was tested using correspondent PCR tests provided by Lytech Ltd., Russia.

Sequencing of the *S. pyogenes* genome was performed using the PromethION platform (Oxford Nanopore Technology, ONT) to obtain long reads and MGI DNBSEQG400 to obtain short reads. The concentration of total DNA was measured using Qubit 4.0 Fluorometer and Qubit dsDNA HS Quantification Assay Kit (Thermo Fisher Scientific, USA). ONT library preparation was performed using the NEBNext ULtra™ II End Repair/dA-Tailing Module (NEB, USA) according to the manufacturer’s protocol. Barcode ligation was performed using Blunt/TA Ligase Master Mix (NEB, USA) and SQK-NBD114.94 (ONT, UK). High-throughput sequencing was performed using GridION with FLO-MIN114 Flow Cell (R10.4.1) and EXP-FLP004 Flow Cell Priming Kit (Oxford Nanopore Technologies, UK). Library preparation for DNBSEQ-G400 sequencing was performed using MGIEasy FS DNA Library Prep Kit (MGI, China) according to the manufacturer’s protocol. Circularization was performed individually for the pooled libraries using MGIEasy Circularization Kit (MGI, China) according to the manufacturer’s protocol. Enzymatic digestion products were pooled and were taken to DNB preparation. Obtained DNB were sequenced on DNBSEQ-G400 sequencer using DNBSEQ-G400RS High-throughput Rapid Sequencing Kit (FCS PE100) (MGI, China) and DNBSEQ-G400RS Rapid Sequencing Flow Cell (MGI, China).

### 2.3 Bioinformatic analysis

The assembly of long reads was carried out using Flye v2.8.1 (5) with further polishing of short reads via Pilon v.1.24.0 (6). Genome annotation was performed using Prokka v1.14.6 (7). Resistance genes were identified using AMRFinderPlus (8) and close inspection of the genome annotation. The sequence type was determined using PubMLST (9). The emm type was determined *in silico* via emmtyper (10).

To analyze 16S rRNA sequencing data, we used the epi2me-labs/wf-metagenomics program (minimap2 + ncbi_16s_18s_28s_ITS database). Visualization performed via python3.

The search for prophage sequences was conducted using Phigaro (11) and VirSorter2 (12). To verify the completeness of the identified prophage sequences, PhageScope (13) was employed.

## 3 Results

The autopsy samples from the patient tested positive for the presence of *S. pyogenes* in all samples and *Metapneumovirus* in samples from the right lung, left lung, and trachea, as determined by RT-PCR. The RT-PCR method identified the presence of *Metapneumovirus* in all 13 nasopharyngeal swab samples collected from contact individuals. Additionally, *Adenovirus* was detected in samples from patients CP8 and CP17, while Epstein–Barr virus was identified in the sample from CP8 and *Rhinovirus* was identified in the sample from CP19. Furthermore, the RT-PCR method identified the presence of *S. pneumoniae* in samples CP4, CP6, CP9, and CP17. The complete set of RT-PCR results is presented in Table 1.

Clinical isolates of *S. pyogenes* were obtained in two replicates (8 total) from all autopsy materials obtained from the patient. Based on the results of phenotypic evaluation, all 8 isolates were found to have similar phenotypic characteristics. The *S. pyogenes* isolate was then cultured and sequenced using both the MGI and Oxford Nanopore platform. This allowed the hybrid assembly of the pathogen to be obtained and subsequently analyzed. Further details of the methodology can be found in the Supplementary material.

To gain further insight into the bacterial microflora, additional V3-V4 region of 16S rRNA sequencing was conducted using the Oxford Nanopore platform on patient samples. The results of the analysis demonstrated the presence of *S. pyogenes* in all autopsy samples from the deceased patient. Additionally, the tracheal tissue sample from the patient was found to contain *Moraxella catarrhalis*, a pathogen that causes respiratory tract infections, primarily in children and less commonly in adults. This pathogen represented approximately 4% of all tracheal infections. Furthermore, approximately 6.4% of the reads in the right lung sample were identified as *Prevotella melaninogenica*, a bacterial species that is a normal representative of the microflora of the upper respiratory tract.

The results of the genomic data analysis, which was conducted using MLST for seven genes (*gki, gtr, murI, mutS, recP, xpt,* and *yqiL*). Based on the results of the typing of all replicates, it was concluded that this *S. pyogenes* strain belongs to ST648. Based on the results of emm-cluster typing, emm-cluster A-C3 and *emm1* type were determined. No specific antibiotic resistance determinants have been identified. However, the genome contains several ABC transporter permeases that may also be associated with antibiotic resistance. A gene for the biosynthesis of streptococcal pyrogenic exotoxin *SpeA*, cysteine proteinase exotoxin *SpeB*, streptococcal pyrogenic exotoxin *SpeJ*, streptococcal pyrogenic exotoxin *SpeG* and streptococcal mitogenic exotoxin *SmeZ*.

It is known from the literature that a number of *S. pyogenes* exotoxins can be encoded by a prophage. Additionally, to check the localization of *S. pyogenes* exotoxin genes, a search for prophage sequences was carried out. Based on the results of this search, the three most reliable prophage candidates were found. Moreover, the *SpeA* gene was discovered near *Sp_prophage_3*, which probably indicates that this gene is transferred specifically by the bacteriophage. However, it is quite difficult to state unequivocally that *SpeA* was transferred to this genome precisely through phage transduction.

## 4 Discussion

Human *Metapneumovirus* (hMPV) is a prevalent infection that affects both children and adults in rare cases. The majority of individuals infected with hMPV do not experience significant complications. However, in some cases, the disease can be difficult for the patient and even lead to death (14). A number of bacterial pathogens are also known to be associated with the severity of the disease. In particular, a double infection caused not only by the virus itself, but also by a bacterial pathogen, can be fatal. Among the works describing dual infections of *Metapneumovirus* and bacterial pathogen, *S. pneumoniae* is very common (2). And in particular, the work (4) showed that *S. pneumoniae* can modulate hMPV infection. Similar cases are also observed in this case, where the presence of hMPV and *S. pneumoniae* simultaneously was also confirmed in the samples of contact persons CP4, CP6, CP9 and CP17. Group A *Streptococcus* (e.g., *S. pyogenes*) was identified in all samples from the patient, but not in those from the contacts. It is probable that the presence of *S. pyogenes* contributed to the patient’s further intoxication, given that it was isolated exclusively from the deceased patient. Conversely, in patients with detected *S. pneumoniae*, intoxication was not observed.

Five genes of the streptococcal exotoxins *SpeA* (15), *SpeB* (16), *SpeJ* (17), *SpeG* (18) and the streptococcal mitogenic exotoxin *SmeZ* (19) were found in the genome of the *S. pyogenes* strain in question. On the other hand, the gene *SpeA* is localized in the neighborhood of the detected *Sp_prophage_3*, which allows to speculate on the subject of phage transduction of this gene. Additionally, previous literature has described instances of *SpeA* gene transfer by T12 phages, which lends further support to the hypothesis that the *SpeA* gene originated as a result of phage transduction in this genome (20, 21). At the same time, the combined effects of various exotoxins could lead to such a severe course of the disease.

The literature also describes the *SpeA* gene as being highly associated with diseases such as scarlet fever and streptococcal toxic shock syndrome (STSS) (22). At the same time, the patient showed signs of severe intoxication, apparently as a result of streptococcal toxic shock syndrome (STSS). It is interesting that the patient had no obvious signs of scarlet fever, and the pathogen itself was observed in many autopsy tissues. There are also cases in the literature where *S. pyogenes* was found in the lungs (23, 24) without scarlet fever signs. Given the presence of *S. pyogenes* in autopsy samples of the spleen, it is reasonable to infer that bacteremia was the initial event, resulting in the pathogen’s entry into the spleen. Another intriguing case from the literature is that of (25), wherein *S. pyogenes* was also identified in the lungs without indications of scarlet fever. This same publication also documented a comparable sequence type, ST648, which is currently under consideration in this article.

The results of the V3-V4 sequencing of the 16S rRNA region also identified *M. catarrhalis*, which is an opportunistic bacterium that causes an inflammatory process in conjunction with other bacterial pathogens of the respiratory tract. In particular, the presence of *M. catarrhalis* can also enhance the adhesion of *S. pyogenes* to human epithelial cells (26). However, in this case, it is impossible to unambiguously confirm the effect of *M. catarrhalis* on *S. pyogenes*, but this fact is quite curious.

A subsequent analysis of the literature revealed that similar cases of bloodstream infections caused by Group A *Streptococcus*, typically provoked by emm1 *S. pyogenes*, were also prevalent in other European countries during 2022 and 2023 (27–31). A similar increase in the incidence of A Streptococcus was observed in the Russian Federation in 2022. Thus, the observed phenomenon was of a broader nature.

In the case considered, the situation was further complicated by hMPV infection. As a result of resistance to hMPV infection and the individual characteristics of the patient’s (eg. genetic immunodeficiency) organism, a decline in immunity occurred, manifested in the form of a strong decrease in the level of neutrophils. As a result, the patient was unable to provide significant resistance to the bacterial pathogen. Moreover, cases of neutropenia in patients with hMPV have also been described in the literature (32). However, it cannot be unequivocally stated that it was hMPV that influenced the drop in neutrophil levels.

The search for antibiotic resistance genes in the *S. pyogenes* strain under consideration did not show any obvious specific determinants of resistance. At the same time, ABC transporter permease genes were discovered in the genome, which can provide nonspecific resistance. However, no obvious determinants of resistance were found during genomic analysis. It is conceivable that had *S. pyogenes* been identified in the patient at an earlier stage and antibiotic treatment commenced, a fatal outcome might have been averted, given the absence of any obvious resistance determinants in the bacterial pathogen.

Antibiotics are usually used for the treatment of *S. pyogenes* (33). Earlier use of antibiotics would probably have inhibited the growth of the bacterial pathogen and bacterial toxin production might have been lower. Early use of amoxicillin and other beta lactam antibiotics here could potentially lead to a better outcome. A case of dual infection with *Metapneumovirus* and *S. pneumoniae* has also been described in the literature (3). The infection in this case was a different bacterial pathogen, but those in this case were treated with empirical antibiotic treatment.

## 5 Conclusion

In the case under consideration, it was challenging to preliminarily identify signs of *S. pyogenes* infection in the patient due to the absence of overt signs of scarlet fever and general bacterial infection. The clinical picture indicated that the infection developed rapidly, a result of STSS. It is important to note that infection with Group A Streptococcus has a more significant impact on children due to the relatively low lethal doses of exotoxin. At the same time, we assume that the strain’s own characteristics, which included as many as 5 exotoxin genes, played an equally important role here.

This case study illustrates the necessity of monitoring for the onset of viral illnesses and colds, as well as the potential for bacterial infections. It is of particular importance to monitor children for the presence of bacterial infections, given that their immune systems are less able to tolerate such infections. The monitoring of bacterial diseases will facilitate the adjustment of antimicrobial use, in order to prevent the widespread overuse of antibiotics.

## Data Availability

The datasets presented in this study can be found in online repositories. The names of the repository/repositories and accession number(s) can be found at: https://www.ncbi.nlm.nih.gov/bioproject/?term=PRJNA1157607, PRJNA1157607.
