# Supplementary material for: Case Report: Fatal case of dual infection Metapneumovirus complicated by Streptococcus pyogenes
Source: Front Med (Lausanne). 2025 Apr 22;12:1576583. doi: 10.3389/fmed.2025.1576583 (PMC12052555; doi:10.3389/fmed.2025.1576583)
Supplement: Supplementary file 1 [file Data_Sheet.docx]

Supplementary Material

# Genome annotation results

The half-resulting assembly and its annotation were additionally visualized in a proxy.


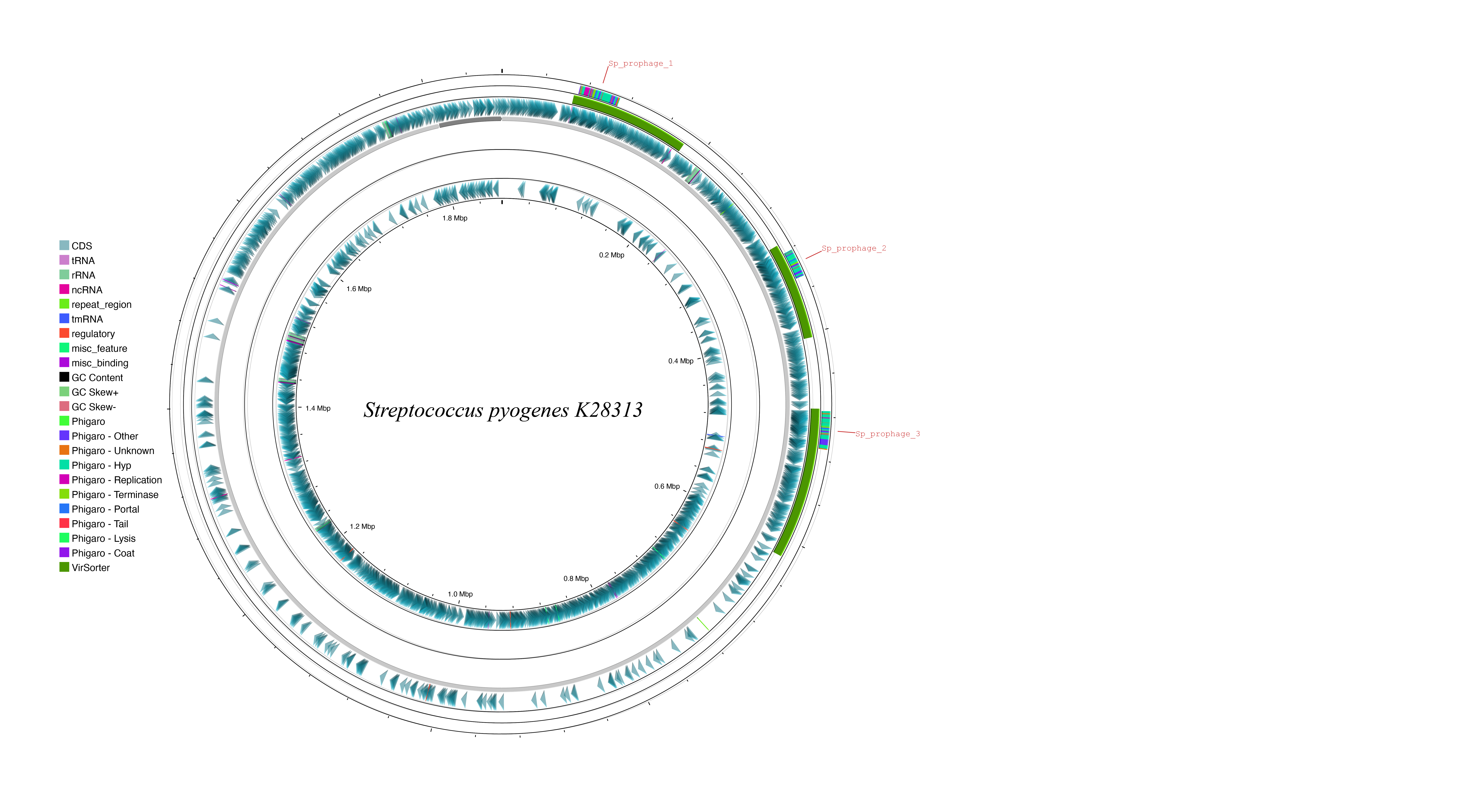


**Supplementary Fig.1** Genome map for *S. pyogenes* assembly (Proksee visualization).

# 16S metagenomic results

We visualized the results of the analysis of 16S rRNA sequencing of samples obtained from the patient in **Supplementary Figure 2** and additionally noted the major organisms in **Supplementary Table 1**.


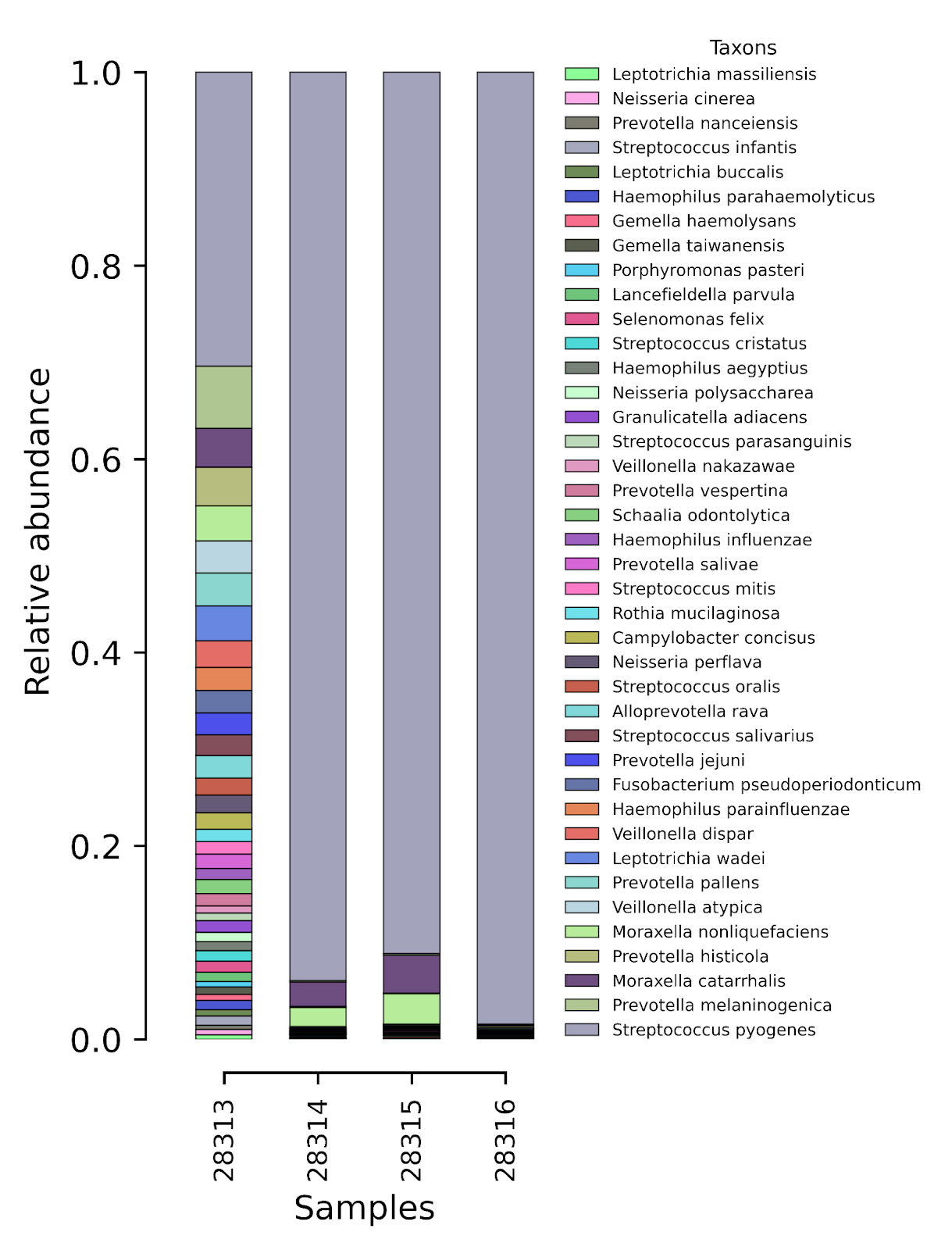


**Supplementary Fig.2** Results of 16S rRNA sequencing of all samples from the patient - right lung (K28313), the left lung (K28314), the trachea (K28315), and the spleen (K28316)

**Supplementary. Table 1.** Major organisms obtained for 16S sequencing results.

| **Sample** | **Source for microbial analysis** | **Major organisms**  **(>5% 16S sequencing)** |
| --- | --- | --- |
| **K28313** | Right lung (tissue) | *Streptococcus pyogenes 30.4%*  *Prevotella melaninogenica 6.4%* |
| **K28314** | Left lung (tissue) | *Streptococcus pyogenes 93.9%* |
| **K28315** | Trachea (tissue) | *Streptococcus pyogenes 91.1%* |
| **K28316** | Spleen (tissue) | *Streptococcus pyogenes 98.4%* |
